# Global Sensitivity Analysis of a Novel Signaling Network for Heart Growth With Local IGF1 Production

**DOI:** 10.1002/cnm.3906

**Published:** 2025-02-09

**Authors:** Christian Bilas, Claus Kratzer, Arne Hinrichs, Andreas Maier, Stephen Wildhirt, Eckhard Wolf, Michael W. Gee

**Affiliations:** ^1^ Mechanics & High Performance Computing Group, TUM School of Engineering and Design, Technical University Munich Garching b. Muenchen Germany; ^2^ Chair for Molecular Animal Breeding and Biotechnology, Gene Center and Department of Veterinary Sciences, Ludwig Maximilians University (LMU) Munich Munich Germany; ^3^ Center for Innovative Medical Models (CiMM), Ludwig Maximilians University (LMU) Munich Oberschleißheim Germany; ^4^ AdjuCor GmbH Munich Germany; ^5^ Laboratory for Functional Genome Analysis (LAFUGA), Gene Center, Ludwig Maximilians University (LMU) Munich Munich Germany

**Keywords:** global sensitivity analysis, heart growth, insulin‐like growth factor 1, signaling network

## Abstract

Signaling networks can be used to describe the dynamic interplay of hormonal and mechanical factors that regulate heart growth. However, a qualitative analysis of signaling networks is often difficult due to their complexity and nonlinear behavior. In this work, a global sensitivity analysis of signaling networks is conducted to determine the most influential factors for heart growth over a range of model inputs. Furthermore, the local production of the hormone insulin‐like growth factor 1 (IGF1) in response to high mechanical stretches as recently described by Zaman et al.(*Immunity*, 54, 2057) and Wong et al.(*Immunity*, 54, 2072) is incorporated. The computational results show that this increases the influence of mechanical stretch on heart growth significantly. Further key influential factors are the hormones norepinephrine (NE), angiotensin II (AngII), and globally produced IGF1 (g‐IGF1). Our sensitivity analysis indicates that the novel consideration of local IGF1 (l‐IGF1) production has to be considered in signaling networks for heart growth.

## Graphical abstract

Signaling networks regulate heart growth through hormonal and mechanical factors, but their complexity makes qualitative analysis challenging. This study's global sensitivity analysis highlights key factors, including the significant impact of local insulin‐like growth factor 1 (IGF1) production in response to mechanical stretches, norepinephrine (NE), angiotensin II (AngII), and globally produced IGF1 (g‐IGF1). Incorporating local IGF1 production is crucial for accurate heart growth modeling.

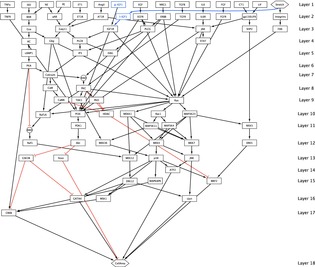

## 1 Introduction

The heart is a dynamic muscular organ that pumps blood throughout the circulatory system. Various factors, whether physiological (such as sports activities, pregnancy, or natural growth and development in pediatric cases) or pathological (including valve defects and heart conditions like dilated cardiomyopathy), can lead to altered pumping demands on the heart [1, 2, 3]. In response, the heart undergoes long‐term adaptations involving changes in its geometry and function. This process is commonly referred to as growth and remodeling (G&R) [4, 5].

On a cellular level, the hormone *insulin‐like growth factor 1* (*IGF1*) plays a crucial role in G&R. When it binds to its receptor *IGF1R*, multiple signaling pathways are activated to regulate cell proliferation, differentiation, metabolism, and survival [6]. *IGF1* is mainly synthesized in the liver and carried to the heart with the bloodstream, causing an endocrine effect [7]. However, it is also produced locally in response to mechanical stretches, causing an autocrine or paracrine effect [8]. Its absence prevents cardiomyocyte growth in hypertension, leading ultimately to heart failure. Thus, locally produced *IGF1* (l‐*IGF1*) is a crucial requirement for heart growth and thus has to be considered in G&R [9].

Signaling networks have been used to represent the complex interplay of mechanical and biochemical stimuli for heart growth on a cellular level [10, 11, 12, 13]. They consist of a set of species and a set of reactions determining the mutual dependencies among species. Species refer to the molecular entities involved in the signaling network, such as proteins, small molecules (e.g., ions, metabolites), nucleic acids (e.g., DNA, RNA), and complexes formed by these molecules. Reactions represent the biochemical interactions or transformations that occur between species within the signaling network, e.g., protein–protein binding. Within these signaling networks, the species *CellArea* has been used to describe heart growth [14].

In the past, signaling networks were modeled with kinetic [15], Boolean [16], fuzzy logic [17], or normalized Hill differential equations [18, 19]. The analysis of these signaling networks is challenging due to the number of parameters and interactions between species. A detailed overview is given by [20].

To analyze the influence of mechanical and biochemical stimuli on the output quantity of interest *CellArea*, sensitivity analysis can be used. In general, one differentiates between local and global sensitivity analysis. Local sensitivity analysis investigates how the model output changes at a fixed set of input values, and it has been applied to signaling networks [10, 18, 21]. In contrast, global sensitivity analysis investigates how the model output changes over a range of inputs [22]. Furthermore, nonlinear effects and interactions between inputs can be analyzed. However, a large number of model evaluations is needed, increasing the computational cost.

In this contribution, the following research objectives are addressed.

We incorporate the local *IGF1* production pathway into an existing signaling network used for modeling cardiac growth based on rather recent findings in [8, 9]. Thus, we choose to base our study on a well‐established and recently published network [14] and demonstrate and discuss changes due to the novel local‐*IGF1* production pathway. Second, we introduce a methodology for computing global sensitivities (GSA), which provides a more comprehensive analysis than local sensitivity approaches. Finally, we apply this methodology to identify the most influential species on heart growth, offering insights that are difficult to obtain using local sensitivity analysis alone.

Our global sensitivity analysis shows that due to the local *IGF1* production, the mechanical influence on heart growth is drastically increased. Furthermore, mechanical stretch, *NE*, *ANGII*, and globally produced *IGF1* (g‐*IGF1*) appeared as the most influential input factors.

## 2 Material and Methods

### 2.1 Signaling Network Modifications

**FIGURE 1:**
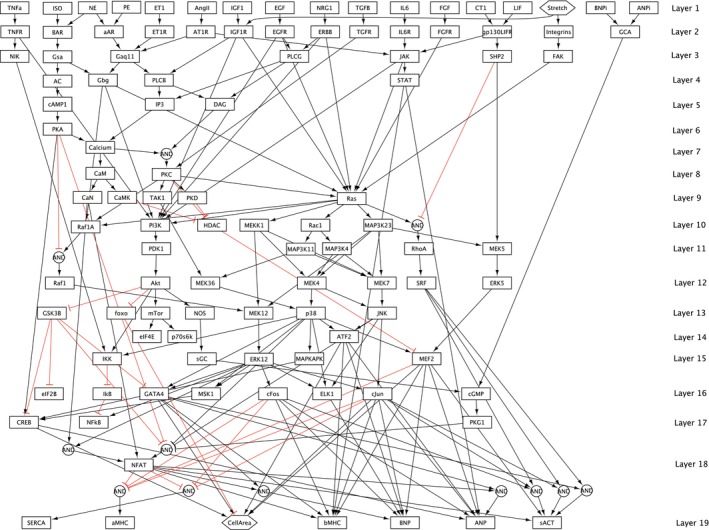
Reference (REF) network containing 106 species and 193 reactions (taken from [10, 14, 21, 23]). It consists of 17 inputs and 7 outputs and is arranged in 19 layers, where all species in a layer depend only on species in previous layers. The species *Stretch* and *CellArea* are represented as hexagons as they are related mechanical quantities. A black connection denotes a species activation, whereas red color represents an inhibition. A detailed list of all species within this network can be found in the appendix (see Table A1).

As a starting point, we utilize an established signaling network originally published by Ryall et al. [21] and subsequently utilized in other studies [10, 14, 23]. For the sake of completeness, this network is shown in Figure 1. It is referred to as the reference (REF) network throughout this paper. The REF network is specifically designed for modeling cardiac growth, making it a suitable reference for our research objectives.

It is important to note that there are other networks, such as presented in the work by Tan et al. [24], which focuses solely on the role of stretch as an input or the work by Saucermann et al. [25], which describes several pathways in cardiac myocytes. Although integrating both networks could yield additional insights, our study concentrates on the recent network presented by Estrada et al. [14].

The input species *Stretch* represents the local mechanical stimulus, and the output species *CellArea* can be used to quantify heart growth [14]. All other species are hormonal and biochemical factors. A detailed list of all species within the network can be found in the appendix (see Table A1).

The REF network is modified in two steps. First, since we are only interested in heart growth, all species and reactions that do not influence *CellArea* are removed. Second, the additional species l‐*IGF1*, accounting for the locally produced *IGF1*, is added into the second layer and is connected to *Stretch* and *IGF1R*. Furthermore, the input species *IGF1* is renamed to g‐*IGF1* as it now only represents the globally produced *IGF1*.

**FIGURE 2:**
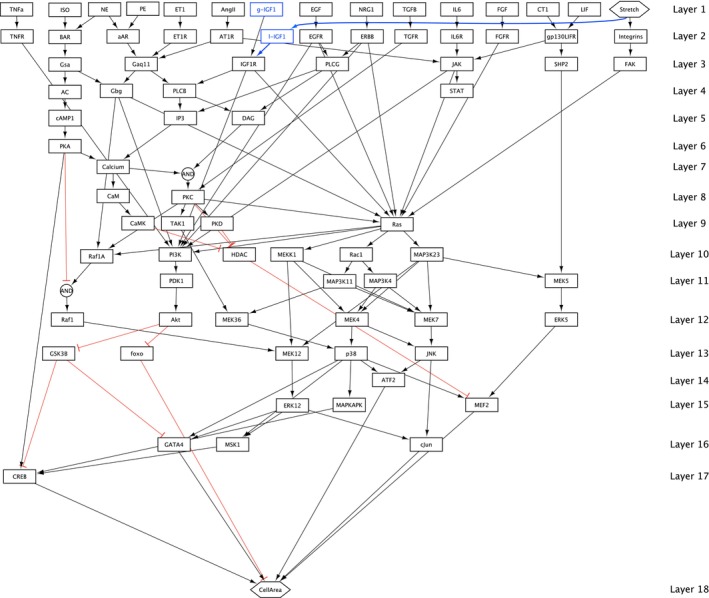
Reduced and modified (R&M) signaling network containing 80 species, 140 reactions, and 18 layers. It consists of 15 input species, *CellArea* remains the only output species. The network modifications are depicted in blue.

The local *IGF1* production is attributed to resident cardiac macrophages, which are in physical contact with cardiomyocytes via focal adhesion complexes. Local *IGF1* production is initiated by a calcium influx through transient receptor potential vanilloid 4 (TRPV4)‐calcium channels, triggering *IGF1* gene expression, as demonstrated in Wong et al. [8]. Research in Zaman et al. [9] has established that locally produced *IGF1* is even a crucial requirement for functional heart remodeling processes. Tissue‐resident macrophages have also other functions as they are involved in tissue development, remodeling, and immune adaption [26]. The obtained novel signaling network is referred to as the reduced and modified (R&M) network and is shown in Figure 2.

### 2.2 Direct Stationary Solution of the Hill Differential Equations

Let S denote the set of all species in a network and ℐ⊂S the set of input species. Each species s∈S is characterized by a concentration $c_{s}\in \left[0,c_{s,\max}\right]$, where $c_{s}=0$ corresponds to the lowest and $c_{s}=c_{s,\max}$ to the highest possible level of activation. In the following, we define a network evaluation $y=f\left(c_{ℐ}\right)$, with $c_{ℐ}=\left\{c_{i}\in \left[0,{\tilde{c}}_{i,\max}\right]\;|\;i\in ℐ\right\}$. In the REF and R&M network, y corresponds to $c_{\text{CellArea}}$.

**FIGURE 3:**
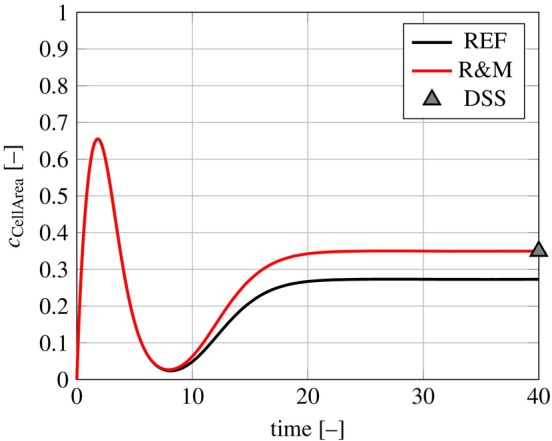
Time‐dependent concentration of species *CellArea* for the REF and R&M network. The direct stationary solution (DSS) is also shown for the R&M network.

To evaluate the REF and R&M networks using Hill differential equations, we choose $c_{s,\max}=1,\tau_{s}=1\;\forall s\in S\backslash ℐ$. If the reader is not familiar with the Hill differential modeling approach, please refer to the Appendix A for an example or see Kraeutler et al. [18]. All reaction weights w are set to 1 [14], and the pseudo time interval is set to $\left[0,40\right]$. To demonstrate the system's behavior under low basal activation under normal in vivo conditions, all input concentrations, including *Stretch*, are assigned to $c_{i}=0.06,\;\forall i\in ℐ$ throughout the entire simulation time [10]. In Figure 3, the time‐dependent concentrations of species *CellArea* are shown for the REF and the R&M network.

In both networks, the concentration $c_{\text{CellArea}}$ approaches a stationary value. The additional pathway from *Stretch* to l‐*IGF1* in the R&M network leads to a higher stationary value of $c_{\text{CellArea}}$.

**FIGURE A4:**
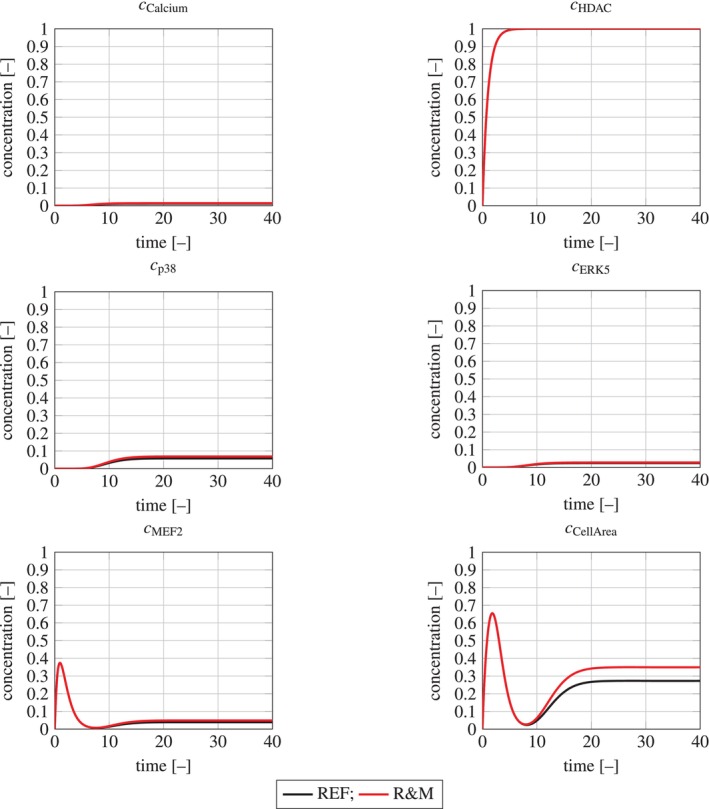
Time‐dependent ODE solutions of the REF and R&M network.

In the following, we consider it sufficient to calculate only the stationary solution of this signaling networks due to the following reasons:

- The observed initial transient oscillations of the concentrations arise from the arbitrarily chosen initial values and  do not allow a statement on heart growth, see Figure A4.
- The timescale of dynamic hormonal changes is significantly smaller than the timescale of heart growth.

Demanding $\frac{{\mathrm{dc}}_{s}}{\mathrm{dt}}=0\;\forall s\in S\backslash ℐ$, the so‐called direct stationary solutions (DSS) are obtained.

As all signaling networks in this paper can be hierarchically arranged in layers, the DSS can be computed using forward substitution consecutively from the first to the last layer. In the Appendix A, a proof that all signaling networks used in this work are asymptotically stable can be found.

#### 2.2.1 Sensitive Input Range

**FIGURE 4:**
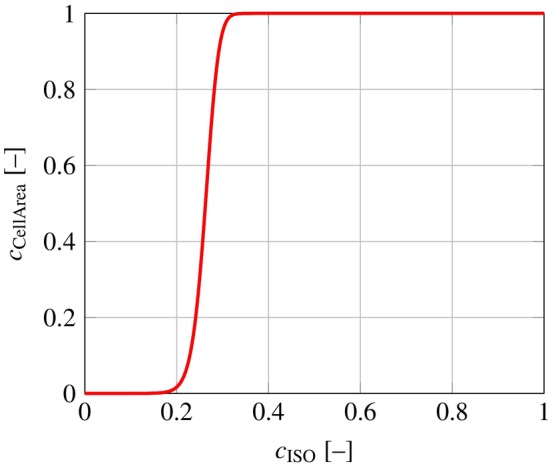
*CellArea* output of the R&M signaling network over a range of $c_{\mathrm{ISO}}$, with all other input concentrations set to 0. For any concentration $c_{\mathrm{ISO}}>0.35$, the output concentration $c_{\text{CellArea}}=1$.

In the REF and R&M signaling networks, a strong monotonic behavior between the inputs $c_{ℐ}$ and the output $c_{\text{CellArea}}$ is observed. A non‐monotonic behavior can only be introduced by inhibitions. Two consecutive inhibitions cancel each other out, see (B4). Within the R&M network, the pathway between *PKA* and *Raf1* is the only pathway, where a single inhibition occurs. However, *PKA* still shows a monotonic behavior with respect to *CellArea*, since it also activates two other pathways. Figure 4 illustrates the concentration $c_{\text{CellArea}}$ over the input concentration $c_{\mathrm{ISO}}$, which activates the *PKA* pathways. Here, all input concentrations are set to 0 except $c_{\mathrm{ISO}}$. Although setting all other input concentrations to zero is not physiological, this approach enables the estimation of an upper bound for input concentrations, where $c_{\text{CellArea}}$ is sensitive to a change of the corresponding input concentration. Notably, even when all other inputs are neglected, $c_{\text{CellArea}}=1$ is reached at $c_{\mathrm{ISO}}>0.35$. This indicates that the upper bound for sensitive input concentrations is ${\tilde{c}}_{i,\max}\ll 1$.

### 2.3 Global Sensitivity Analysis

The REF and R&M signaling networks show nonlinear effects due to the nonlinearity of the Hill activation function and the various interactions between the species. Thus, global sensitivity measures have to be applied. Global sensitivity analysis is preferred over local sensitivity analysis as it captures the interactions and nonlinearities across the entire parameter space, providing a more comprehensive understanding of the system's behavior.

A global sensitivity measure evaluates the sensitivity of the function output over a range of input values. Under certain orthogonality assumptions, $c_{\text{CellArea}}=f\left(c_{ℐ}\right)$ can be decomposed into its mean value $f_{0}$, first‐order effects, and interaction terms [22, 27]

(1) $$f\left(c_{ℐ}\right)=f_{0}+\underset{\text{first order}}{\underbrace{\sum_{i}f_{i}\left(c_{i}\right)}}+\underset{\text{second order}}{\underbrace{\sum_{i<j}f_{\mathrm{ij}}\left(c_{i},c_{j}\right)}}+\ldots +\underset{n^{\mathrm{th}}\;\text{order}}{\underbrace{f_{1\ldots n}\left(c_{1},\ldots ,c_{n}\right)}},$$

where $f_{i}$ depends solely on $c_{i}$, $f_{\mathrm{ij}}$ on $c_{i}$ and $c_{j}$, etc. [27]. In case the summands in (1) are uncorrelated, the total variance $V_{\mathrm{tot}}$ of the model output is

(2) $$V_{\mathrm{tot}}=\sum_{i}V_{i}\left(f_{i}\left(c_{i}\right)\right)+\sum_{i<j}V_{\mathrm{ij}}\left(f_{\mathrm{ij}}\left(c_{i},c_{j}\right)\right)+\ldots +V_{1\ldots n}\left(f_{1\ldots n}\left(c_{1},\ldots ,c_{n}\right)\right),$$

where the variances are defined as [28]

(3) $$V_{i}\left(f_{i}\left(c_{i}\right)\right)=V_{c_{i}}\left(E_{c_{∼i}}\left(c_{\text{CellArea}}|c_{i}\right)\right),$$

(4) $$V_{\mathrm{ij}}\left(f_{\mathrm{ij}}\left(c_{i},c_{j}\right)\right)=V_{c_{i},c_{j}}\left(E_{c_{∼i,j}}\left(c_{\text{CellArea}},c_{i}|c_{j}\right)\right)-V_{i}\left(f_{i}\left(c_{i}\right)\right)-V_{j}\left(f_{j}\left(c_{j}\right)\right),$$

and analogously for higher order terms. Here, $E_{c_{∼i}}\left(c_{\text{CellArea}}|c_{i}\right)$ is the expectation of $c_{\text{CellArea}}$ when $c_{i}$ is fixed and $V_{c_{i}}$ is the variance of $c_{\text{CellArea}}$ for the input concentration $c_{i}$. The expression ∼i denotes that all inputs in ℐ except the ith input are varied in the computation of the mean value. Equations (1) and (2) yield the ANOVA (analysis of variance) decomposition [29, 30]. With that, Sobol indices as global sensitivity estimators can be defined [28].

### 2.4 Sobol Indices

The first‐order sensitivity index

(5) $$S_{i}=\frac{V_{c_{i}}\left(E_{c_{∼i}}\left(c_{\text{CellArea}}|c_{i}\right)\right)}{V_{\mathrm{tot}}\left(c_{\text{CellArea}}\right)},$$

measures the first‐order contribution of $c_{i}$, accounting for the reduction in the variance of $c_{\text{CellArea}},$ relative to the entire output variance [28].

The total‐order index

(6) $$T_{i}=\frac{E_{c\sim_{i}}\left(V_{c_{i}}\left(c_{\text{CellArea}}|c\sim_{i}\right)\right)}{V_{\mathrm{tot}}\left(c_{\text{CellArea}}\right)}=1-\frac{V_{c_{∼i}}\left(E_{c{∼}_{i}}\left(c_{\text{CellArea}}|c_{\sim i}\right)\right)}{V_{\mathrm{tot}}\left(c_{\text{CellArea}}\right)},$$

measures the first and higher order influences of input $c_{i}$ and interaction terms on $c_{\text{CellArea}}$ [31]. If $T_{i}\approx 0$, $c_{i}$ has a negligible contribution to the total variance $V_{\mathrm{tot}}$.

### 2.5 Computation of Sobol Indices

As the evaluation of (5) and (6) in case of many model inputs is computationally expensive, variances are estimated with the Monte Carlo (MC) method [32] using random or quasi‐random numbers (e.g., Latin Hypercube sampling, Sobol’ quasi‐random numbers) [33, 34]. For that, the so‐called base sample matrices A and $B\in {\mathbb{R}}^{N_{I}\times N_{S}}$ are introduced and each matrix is sampled individually and independent from the respective other. Exemplarily, the matrix A reads

(7) $$A=\left[\begin{array}{cc} c_{11} & \cdots \\ \vdots & \\ c_{n1} & \cdots \\ \vdots & \\ c_{N_{S}1} & \cdots \end{array}\right.\underset{i^{\mathrm{th}}\text{input}}{\underbrace{\begin{array}{c} c_{1i}\\ \vdots \\ c_{\mathrm{ni}}\\ \vdots \\ c_{N_{S}i} \end{array}}}\left.\begin{array}{cc} \cdots & c_{1N_{I}}\\ & \vdots \\ \cdots & c_{nN_{I}}\\ & \vdots \\ \cdots & c_{N_{S}N_{I}} \end{array}\right]\begin{array}{c} \}n^{\mathrm{th}}\text{sample} \end{array}.$$

where $N_{I}$ is the number of inputs, and $N_{S}$ is the number of samples [35]. In addition, the matrix $A_{B}^{\left(i\right)}$ is formed, which consists of all columns from A except the $i^{\mathrm{th}}$ column which is from B. The matrix $B_{A}^{\left(i\right)}$ is formed accordingly. With these matrices, sensitivity estimators to compute $S_{i}$ and $T_{i}$ can be defined, see Table 1. These estimators are based on the work of Sobol [28, 36] (Sob), Saltelli [22] (Sat), Jansen [22, 27] (Jan), and Homma [31] (Hom). Therein, $f{\left(A\right)}_{j}$ denotes a network evaluation, where the $j^{\mathrm{th}}$ column of A is used as input concentrations. Similarly, the function evaluations $f{\left(B\right)}_{j}$, $f{\left(B_{A}^{\left(i\right)}\right)}_{j}$, and $f{\left(A_{B}^{\left(i\right)}\right)}_{j}$ are defined.

**TABLE 1:** Estimators to compute first‐order $S_{i}$ and total‐order $T_{i}$ sensitivities for the network function $c_{\text{CellArea}}=f\left(c_{ℐ}\right)$ with mean value $f_{0}$ and number of samples $N_{S}$.

$S_{i}$, see Equation (5)	References
$S_{i}^{\mathrm{Sob}}=\frac{1}{N_{S}}\sum_{j=1}^{N_{S}}\left[f{\left(A\right)}_{j}f{\left(A_{B}^{\left(i\right)}\right)}_{j}-f_{0}^{2}\right]$	[28]
$S_{i}^{\mathrm{Sat}}=\frac{1}{N}\sum_{j=1}^{N_{S}}\left[f{\left(B\right)}_{j}\left(f{\left(A_{B}^{\left(i\right)}\right)}_{j}-f{\left(A\right)}_{j}\right)\right]$	[22]
$S_{i}^{\mathrm{Jan}}=V_{\mathrm{tot}}\left(c_{\text{CellArea}}\right)-\frac{1}{2N_{S}}\sum_{j=1}^{N_{S}}{\left[f{\left(A\right)}_{j}-f{\left(B_{A}^{\left(i\right)}\right)}_{j}\right]}^{2}$	[27]

$T_{i}$, see Equation (6)	
$T_{i}^{\mathrm{Hom}}=\frac{1}{N_{S}}\sum_{j=1}^{N_{S}}\left[f{\left(A\right)}_{j}\left(f{\left(A\right)}_{j}-f{\left(A_{B}^{\left(i\right)}\right)}_{j}\right)\right]$	[31]
$T_{i}^{\mathrm{Sob}}=\frac{1}{N_{S}}\sum_{j=1}^{N_{S}}\left[f{\left(A\right)}_{j}\left(f{\left(A\right)}_{j}-f{\left(A_{B}^{\left(i\right)}\right)}_{j}\right)\right]$	[36]
$T_{i}^{\mathrm{Jan}}=\frac{1}{2N_{S}}\sum_{j=1}^{N_{S}}{\left[f{\left(A\right)}_{j}-f{\left(A_{B}^{\left(i\right)}\right)}_{j}\right]}^{2}$	[22, 27]

The computational cost to evaluate the network using all samples of matrix A or B scales with $N_{S}$. To evaluate the network for all matrices $A_{B}^{\left(i\right)}$ or $B_{A}^{\left(i\right)}$ with $i=1,..,N_{S}$, the computational cost scales with $N_{I}\cdot N_{S}$. When using DSS, the required model evaluations are computationally cheap, and thus global sensitivity analysis becomes affordable.

## 3 Global Input Sensitivity Results

As previously discussed, for too large input concentrations the output concentration $c_{\text{CellArea}}=1$. In that case, a very large number of input samples would be needed to obtain meaningful sensitivity estimates. For the choice ${\tilde{c}}_{i,\max}=0.12$ meaningful sensitivity estimates are obtained.

**FIGURE 5:**
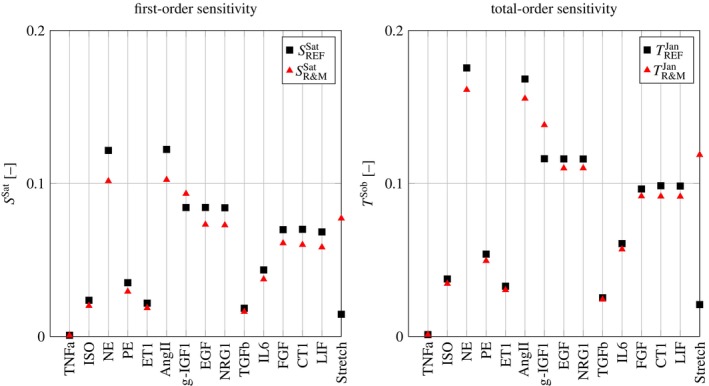
First‐ and total‐order sensitivities of $c_{\text{CellArea}}$ with respect to all input species in the REF and R&M network.

The base sample matrices A and B thus are filled using Latin Hypercube sampling [37] on the interval $\left[0,0.12\right]$ for all inputs. Exemplarily, the sensitivity indices $S^{\mathrm{Sat}}$ and $T^{\mathrm{Jan}}$ of *CellArea* with respect to the input species are computed for the REF and R&M network using $N_{S}=1\cdot 10^{6}$. Results are shown in Figure 5.

Compared to the REF network, both sensitivity indices of *Stretch* increase approximately by a factor of five for the R&M network. Hence, in the R&M network, together with g‐*IGF1*, *NE*, and *ANGII*, *Stretch* becomes one of the most influential factors on *CellArea*.

The additional pathway from *Stretch* to *IGF1R* induces an additional interaction between the inputs g‐*IGF1* and *Stretch* in the R&M network. Hence, the total‐order sensitivity of g‐*IGF1* increases. Both sensitivities of *TNFa* are small because it only enters one OR connection with six other species. Furthermore, the sensitivities of *CT1* and *LIF* are roughly the same as both input species are only connected to *gp130LIFR*. Also, due to the increased sensitivity with respect to *Stretch*, the remaining sensitivities decrease.

**FIGURE 6:**
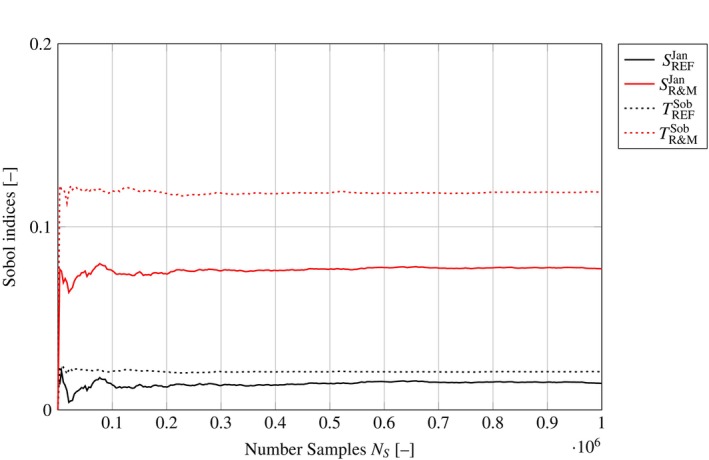
Sobol indices for *CellArea* with respect to *Stretch* within the REF and R&M network plotted over the number of samples.

**FIGURE A6:**
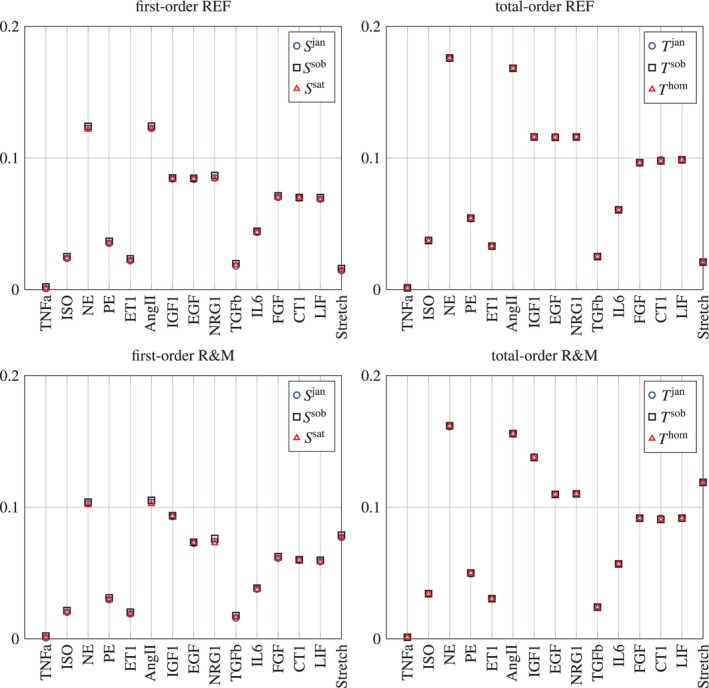
Sensitivity indices of *CellArea* with respect to the input concentrations for the REF and R&M network. The sensitivity analysis results for the REF network are displayed in the top row, while the bottom row showcases the sensitivities for the R&M network.

Figure 6 shows the first‐ and second‐order estimators for both networks for the input node *Stretch* over the number of samples used for their computation, demonstrating convergence of the sensitivity analysis. All six estimators from Table 1 are shown in the appendix Figure A6. A numeric representation of the sensitivities can be found in the appendix in Tables A2 and A3.

## 4 Discussion

Based on findings in Zaman et al. [9] and Wong et al. [8], we incorporated mechanically induced *IGF1* production into an existing signaling network for heart growth [21] by adding an additional pathway from *Stretch* and l‐*IGF1*. Furthermore, we removed all species and reactions that do not influence *CellArea*, making the analysis of the network much cheaper. While we focused on integrating strain‐ induced *IGF1* production into the established signaling network for heart growth [21], it is important to note that other networks can also be utilized. For instance, incorporating additional connections from the work of Tan et al. [24] could provide further insights into the role of stretch as an input. However, it is crucial to emphasize that global sensitivity analysis can be applied to any network and across all nodes, allowing for a comprehensive evaluation of influential factors and their interactions.

Our sensitivity analysis for the species *CellArea*, see Figure 5, shows that within the original REF network, *Stretch* has only a minor contribution to heart growth compared to other influential quantities. However, it has been shown that Stretch is most important for cardiomyocytes in heart growth [38]. Due to the additional pathway, *Stretch* became one of the most influential factors for growth within the R&M network. The sensitivities of *CellArea* with respect to *Stretch* are approximately five times higher than in the REF network.

All presented signaling networks converge to their stationary solution. Transient effects due to arbitrary initial conditions are not physiologically interpretable, see Figure 3. Furthermore, choosing appropriate initial conditions and reaction time constants is challenging due to the difficulty in data acquisition. For a fixed set of parameters, the timescale of hormonal and biochemical changes is smaller compared to the timescale of heart growth. This motivated the DSS approach, where reaction time constants and initial conditions are no longer required.

Given that the considered signaling networks are nonlinear with numerous interactions, we performed a global sensitivity analysis to identify the most influential factors on *CellArea*. The computational cost for many model evaluations is reduced by the DSS approach. We estimated the first‐ and total‐order sensitivities by using a variance‐based Monte Carlo approach. For sampling the input concentrations, we identified a physiological input range.

The first‐order sensitivity index showed an increased sensitivity of *Stretch* and decreased sensitivities of all other species for the R&M network. The total‐order sensitivity, which accounts for interactions between the species, increased for *Stretch* and g‐*IGF1* while decreasing for all other species.

The most influential factors on *CellArea* were identified as *Stretch*, *NE*, *AngII*, and g‐*IGF1*. It has been shown that locally produced *IGF1* is necessary in physiological heart growth [6, 8, 9]. Moreover, the species *NE* holds pivotal significance in myocytes and various cell types within the heart, governing crucial biological functions including growth [39, 40]. Additionally, *AngII* modulates cardiac contractility, cell coupling, and impulse propagation and contributes to cardiac growth and remodeling [41, 42].

Our sensitivity analysis revealed that the species *TNFa* exerts the least influence on heart growth. Previous studies have demonstrated that during physiological heart growth, the *TNFa* signaling cascade may not be necessary for normal cardiogenesis or that it could be redundant and compensated for by alternative signaling pathways [43]. However, in pathological conditions, the concentration of circulating *TNFa* can be elevated [44] and can induce hypertrophy [45]. These findings align with the outcomes of our sensitivity analysis.

## 5 Outlook

The calibration of signaling networks is challenging due to the high number of inputs and model parameters. However, in a so‐called factor fixing [35] approach, the presented results can be used to reduce the dimensionality of the input space and calibrate the signaling network.

Similar to the framework presented in [14], the proposed R&M signaling network could be coupled to a mechanical finite element model of the heart. For that, transfer functions to map parameters between the mechanical model and the signaling network need to be defined. One transfer function maps a strain metric from the mechanical model to the normalized concentration of Stretch. A possible definition could be based on the global longitudinal strain defined as the maximal contraction during systole. Physiological baseline values and pathological cases are reported in [46] and [47]. Another transfer function maps the output concentration of *CellArea* back to a physically interpretable growth rate value. This transfer function could be based on the physiological mass increase per year [48].

## Ethics Statement

The authors have nothing to report.

## Conflicts of Interest

The authors declare no conflicts of interest.

## Figures and Tables

**FIGURE A1:**
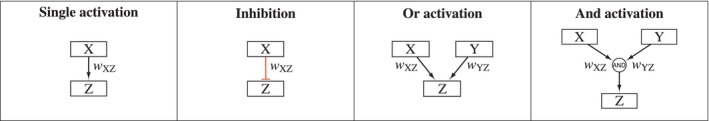
Different reaction types within a signaling network with reaction weight w. In a single activation, species X activates species Z, whereas in an inhibition, species X inhibits the activation of species Z. In an OR activation, either species X or Y can activate Z, and in an AND activation, both species X and Y must be active to activate species Z.

**FIGURE A2:**
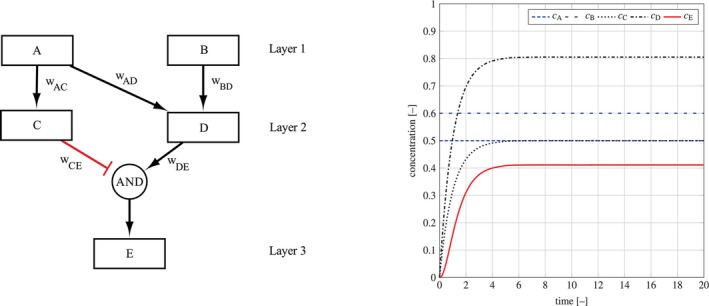
The demonstrator network, illustrated on the left, consists of five species $S=\left\{A,B,C,D,E\right\}$ with $ℐ=\left\{A,B\right\}$. On the right, the time‐dependent solutions of the corresponding Hill differential equations are depicted. For constant input concentrations $c_{A}=0.5$ and $c_{B}=0.6$, the species C and D are activated and concentrations rise until a stationary value is reached. Subsequently, species E, inhibited by C, attains a stationary concentration $c_{E}=0.41$.

**FIGURE A3:**
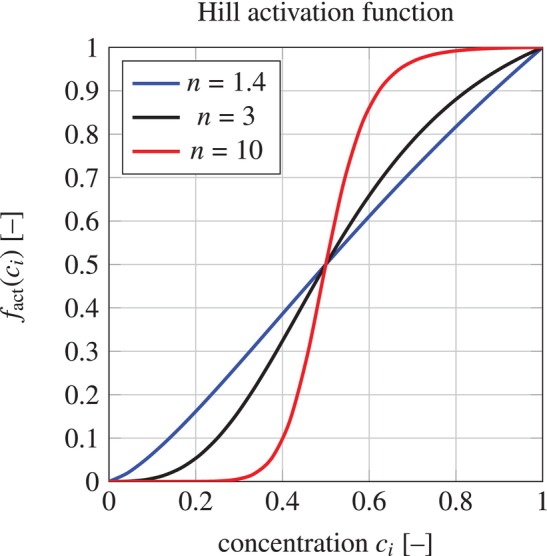
Nonlinear Hill activation function with w=1 and ${\mathrm{EC}}_{50}=0.5$.

**FIGURE A5:**
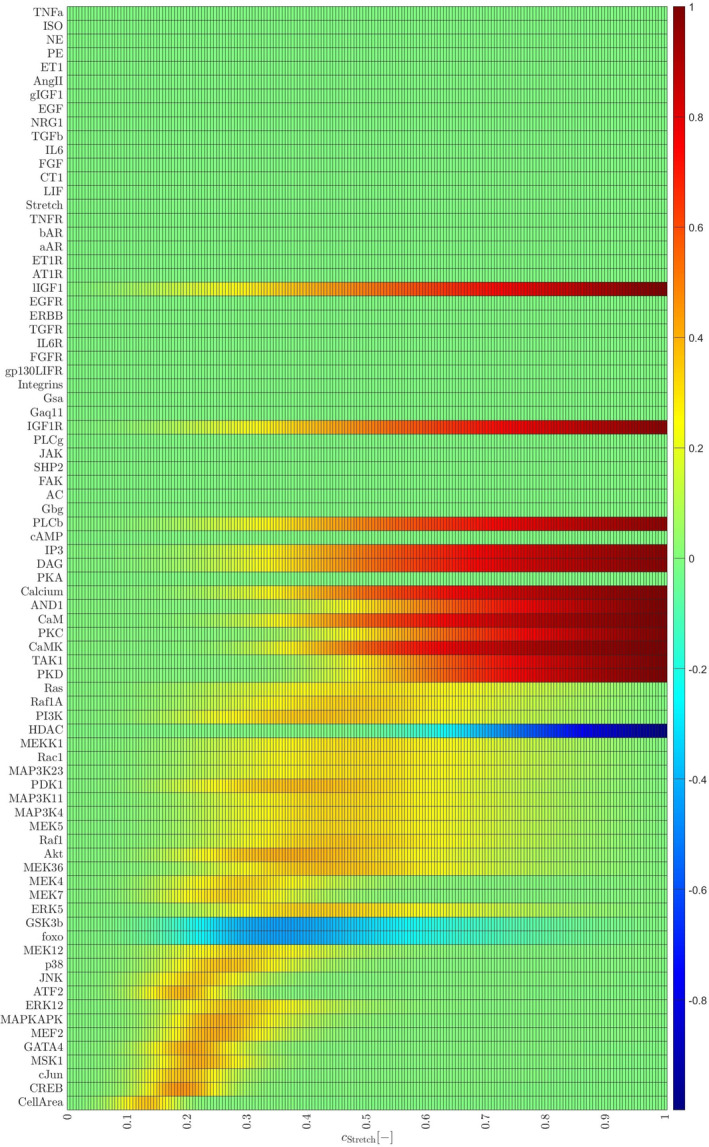
Heatmap illustrating the difference in stationary concentrations between the R&M and REF networks. Input concentrations are set to $c_{i}=0.06,\;\forall i\in ℐ$, except for $c_{\text{Stretch}}$, which varies from 0 to 1. The y‐axis displays all species within both networks. Positive values indicate elevated concentrations in the R&M network, while negative values denote a decreased concentration.

## Data Availability

All relevant data is included in the manuscript itself. The manuscript includes an appendix.

## Appendix A

### Signaling Network Species

**TABLE A1:** List of all species within the REF and R&M network. Nodes that do not interact with CellArea are marked with a *.

Abbreviations	Name	Layer
TNFa	Tumor necrosis factor	Layer 1
ISO	Isoproterenol	
NE	Norepinephrine	
PE	Phycoerythrin	
ET1	Endothelin‐1	
AngII	Angiotensin ii	
g‐IGF1	Global insulin‐like growth factor	
EGF	Epidermal growth factor	
NRG1	Neuregulin 1	
TGFb	Transforming growth factor beta	
IL6	Interleukin 6	
FGF	Fibroblast growth factor	
CT1	Cardiotrophin 1	
LIF	Eukemia inhibitory factor	
Stretch	Mechanical input	
BNPi*	Brain natriuretic peptide input	
ANPi*	Atrial natriuretic factor input	
TNFR	Tumor necrosis factor receptor	Layer 2
bAR	*β*‐adrenergic receptor	
aAR	*α* adrenergic receptor	
ET1R	Endothelin‐1 receptor	
AT1R	Angiotensin II receptor	
l‐IGF1	Local insulin‐like growth factor	
EGFR	Epidermal growth factor receptor	
ERBB	Erythroblastic leukemia viral oncogene homolog 2 and 3 or 4	
TGFR	Transforming growth factor teceptors	
IL6R	Interleukin 6 receptor and interleukin 6 signal transducer gp130	
FGFR	Fibroblast growth factor receptor	
gp130LIFR	Leukemia inhibitory receptor alpha and interleukin 6 signal transducer	
Integrins	Integrins	
GCA*	Guanylate cyclase A	
NIK*	NFkB inducing kinase	Layer 3
Gsa	G protein alpha s	
Gaq11	G protein alpha subunit q or 11	
IGF1R	Insulin‐like growth factor receptor	
PLCg	Phopholipase C gamma 1	
JAK	Janus kinase 1 or 2	
SHP2	Protein tyrosine phosphatase, non‐receptor type 11	
FAK	Focal adhesion kinase	
AC	Adenylyl cyclase	Layer 4
Gbg	G protein beta and gamma subunits	
PLCb	Phospholipase C beta	
STAT*	STAT	
cAMP	Cyclic AMP	Layer 5
IP3	Inositol triphosphate	
DAG	Diacylglycerol	
PKA	Protein kinase A	Layer 6
Calcium	Calcium	Layer 7
CAM	Calmodulin	Layer 8
PKC	Protein kinase C	
CaN*	Calcineurin	Layer 9
CaMK	CaM kinase	
TAK1	TGF‐beta activated kinase 1	
PKD	Protein kinase d	
Ras	Rat sarcoma viral oncogene homolog	
Raf1A	Activated raf1	Layer 10
PI3K	Phosphatidyl inositol 3 kinase	
HDAC	Histone deacetylase	
MEKK1	MAPK kinase kinase 1	
Rac1	Ras‐related C3 botulinum toxin substrate 1	
MAP3K23	MAPK kinase kinase 2 or 3	
PDK1	3‐phosphoinositide dependent protein kinase‐1	Layer 11
MAP3K11	MAPK kinase kinase 11	
MAP3K4	MAPK kinase kinase 4	
RhoA*	Ras homolog gene family, member A	
MEK5	MAPK kinase 5	
Raf1	Raf1	Layer 12
Akt	Protein kinase B	
MEK36	MAPK kinase 3 or MAPK kinase 6	
MEK4	MAPK kinase 4	
MEK7	MAPK kinase 7	
SRF*	Serum response factor	
ERK5	ERK5	
GSK3b	Glycogen synthase kinase 3 beta	Layer 13
foxo	Forkhead box O	
mTor*	Mechanistic target of rapamycin	
NOS*	Endothelial nitric oxide synthase	
MEK12	MAPK kinase 1 or MAPK kinase 2	
p38	p38 mitogen‐activated protein kinase	
JNK	c‐Jun N‐terminal kinase	
elF4E*	Eukaryotic translation initiation factor 4E	Layer 14
p70s6k*	70 kDa ribosomal protein S6 kinase I	
ATF2	Activating transcription factor 2	
IKK*	Inhibitor of kappa light polypeptide gene enhancer in B‐cells, kinase beta	Layer 15
sGC*	Soluble guanylyl cyclase	
ERK12	Extracellular signal‐regulated kinases 1 or 2	
MAPKAPK	MAPK‐activated protein kinase	
MEF2	Myocyte enhancer factor‐2	
elF2B*	Eukaryotic initiation factor 2	Layer 16
IkB*	Nuclear factor of kappa light polypeptide gene enhancer in B‐cells inhibitor	
GATA4	Protein GATA	
MSK1	Ribosomal protein S6 kinase, 90 kDa, polypeptide 5	
cFos*	Protein c‐Fos	
ELK1*	ELK1	
cJun	Protein cJun	
cGMP*	Cyclic guanosine monophosphate	
CREB	cAMP response element binding	Layer 17
NFkB*	Nuclear factor kappa‐light‐chain‐enhancer of activated B cells	
PKG1*	cGMP‐dependent protein kinase 1	
NFAT*	Nuclear factor of activated T‐cells	Layer 18
SERCA*	Sarcoplasmic reticulum	Layer 19
*α*MHC*	*α*‐myosin heavy chain	
CellArea	Growth target node	
*β*MHC*	*β*‐myosin heavy chain	
BNP*	Brain natriuretic peptide	
ANP*	Atrial naturetic peptide	
sACT*	Skeletal *α*‐Actin	

### Hill Differential Equation Approach Details

For the sake of completeness and readability, we briefly repeat fundamentals of network modeling using Hill differential equations. This section is mainly a summary of the work published in Kraeutler et al. [18].

There are four different reaction types in signaling networks: single activation, inhibition, AND activation, and OR activation. A graphical representation of these four reaction types is given in Figure A1.

Let X,Y,Z∈S be species in a signaling network. The Hill activation function $f_{\mathrm{act}}:\left[0,1\right]\mapsto \left[0,1\right]$ from species X to Z is defined by

(A1)
$$f_{\mathrm{act}}^{\to Z}\left(c_{X}\right)≔w_{\mathrm{XZ}}\frac{{\mathrm{Bc}}_{X}^{n}}{K^{n}+c_{X}^{n}},$$

where $w_{\mathrm{XZ}}\in \left[0,1\right]$ is the reaction weight, $B=\frac{{\mathrm{EC}}_{50}^{n}-1}{2{\mathrm{EC}}_{50}^{n}-1}$, $K={\left(B-1\right)}^{\frac{1}{n}}$, ${\mathrm{EC}}_{50}=0.5$ and n=1.4 [18]. The Hill coefficient n determines the steepness of the Hill curve (see Figure A3 in the appendix). ${\mathrm{EC}}_{50}$ is the input species concentration required for half‐maximal activation of an output species. Both parameters K and B ensure that

(A2)
$$f_{\mathrm{act}}\left(1\right)=1,$$

(A3)
$$f_{\mathrm{act}}\left({\mathrm{EC}}_{50}\right)=0.5,$$

see Kraeutler et al. [18]. The inhibition function $f_{\mathrm{inh}}:\left[0,1\right]\mapsto \left[0,1\right]$ from species X to Z is defined by

(A4)
$$f_{\mathrm{inh}}^{\to Z}\left(c_{X}\right)≔1-f_{\mathrm{act}}^{\to Z}\left(c_{X}\right).$$

The logical functions $\text{OR}:\left[0,1\right]\times \left[0,1\right]\mapsto \left[0,1\right]$ and $\text{AND}:\left[0,1\right]\times \left[0,1\right]\mapsto \left[0,1\right]$ between species X, Y, and Z are defined by

(A5)
$$\text{OR}\left(f_{\mathrm{act}}^{\to Z}\left(c_{X}\right),f_{\mathrm{act}}^{\to Z}\left(c_{Y}\right)\right)≔f_{\mathrm{act}}^{\to Z}\left(c_{X}\right)+f_{\mathrm{act}}^{\to Z}\left(c_{Y}\right)-f_{\mathrm{act}}^{\to Z}\left(c_{X}\right)f_{\mathrm{act}}^{\to Z}\left(c_{Y}\right),$$

(A6)
$$\text{AND}\left(f_{\mathrm{inh}}^{\to Z}\left(c_{X}\right),f_{\mathrm{act}}^{\to Z}\left(c_{Y}\right)\right)≔f_{\mathrm{inh}}^{\to Z}\left(c_{X}\right)\;f_{\mathrm{act}}^{\to Z}\left(c_{Y}\right).$$

In case of more than two inputs, as in the REF and R&M network, the AND and OR operations are applied recursively. Exemplary, for three inputs, one has

(A7)
$$\text{AND}\left(\cdot ,\cdot ,\cdot \right)≔\text{AND}\left(\cdot ,\text{AND}\left(\cdot ,\cdot \right)\right),$$

(A8)
$$\text{OR}\left(\cdot ,\cdot ,\cdot \right)≔\text{OR}\left(\cdot ,\text{OR}\left(\cdot ,\cdot \right)\right).$$

#### Demonstrator network

For illustration purposes, a demonstrator network is introduced, see Figure A2. It contains all four types of reactions. Following Kraeutler et al. [18], the Hill differential equations for the demonstrator network are

(A9)
$$\frac{{\mathrm{dc}}_{C}}{\mathrm{dt}}=\frac{1}{\tau_{C}}\left(f_{\mathrm{act}}^{\to C}\left(c_{A}\right)\;c_{C,\max}-c_{C}\right),$$

(A10)
$$\frac{{\mathrm{dc}}_{D}}{\mathrm{dt}}=\frac{1}{\tau_{D}}\left(\text{OR}\left(f_{\mathrm{act}}^{\to D}\left(c_{A}\right),f_{\mathrm{act}}^{\to D}\left(c_{B}\right)\right)\;c_{D,\max}-c_{D}\right),$$

(A11)
$$\frac{{\mathrm{dc}}_{E}}{\mathrm{dt}}=\frac{1}{\tau_{E}}\left(\text{AND}\left(f_{\mathrm{inh}}^{\to E}\left(c_{C}\right),\;f_{\mathrm{act}}^{\to E}\left(c_{D}\right)\right)\;c_{E,\max}-c_{E}\right).$$

The parameters $\tau_{C}$, $\tau_{D}$, and $\tau_{E}$ are reaction time constants and are chosen to $\tau_{s}=1,\;\forall s\in \left\{C,D,E\right\}$. They influence the dynamics of the corresponding activation. Constant input concentrations $c_{A}=0.5$ and $c_{B}=0.6$ are chosen. The remaining species are initialized to $c_{s}=0,\;\forall s\in S\backslash ℐ$. The maximal activation is set to $c_{s,\max}=1,\;\forall s\in S$ and all reaction weights are w=1. The system of ordinary differential Equations ((A9), (A10), (A11)) is numerically solved with a third‐order explicit Runge–Kutta method [49] on the pseudo time interval $t\in \left[0,20\right]$. The numerical solution of the system is shown in Figure A2 on the right.

For the demonstrator network, derived from (B9)–(B11), the resulting DSS are

(A12)
$$c_{C}^{\mathrm{DSS}}=f_{\mathrm{act}}\left(c_{A}\right)c_{C,\max},$$

(A13)
$$c_{D}^{\mathrm{DSS}}=\text{OR}\left(f_{\mathrm{act}}\left(c_{A}\right),f_{\mathrm{act}}\left(c_{B}\right)\right)c_{D,\max},$$

(A14)
$$c_{E}^{\mathrm{DSS}}=\text{AND}\left(f_{\mathrm{inh}}\left(c_{C}\right),f_{\mathrm{act}}\left(c_{D}\right)\right)c_{E,\max}.$$

#### Activation function and its derivatives

The derivatives of all different reaction types are defined as

(A15)
$$\begin{array}{l} \text{Single activation to}\;Y\;\text{for input}\;X\\ \;\frac{{\mathrm{dc}}_{Y}}{{\mathrm{dc}}_{X}}=c_{Y,\max}\;\frac{{\mathrm{df}}_{\mathrm{act}}^{\to Y}\left(c_{X}\right)}{{\mathrm{dc}}_{X}}, \end{array}$$

(A16)
$$\begin{array}{l} \text{And activation to}\;Z\;\text{for}\;N\;\text{inputs}\\ \;\frac{{\mathrm{dc}}_{Z}}{{\mathrm{dc}}_{X}}=c_{Z,\max}\;\frac{{\mathrm{df}}_{\mathrm{act}}^{\to Z}\left(c_{X}\right)}{{\mathrm{dc}}_{X}}\;\prod_{I}^{N}f_{\mathrm{act}}^{\to Z}\left(c_{I}\right)\;\text{with}\;I\in S\backslash \left\{X\right\}, \end{array}$$

(A17)
$$\begin{array}{l} \text{Oractivation to}\;Z\;\text{for}\;N\;\text{inputs}\\ \;\frac{{\mathrm{dc}}_{Z}}{{\mathrm{dc}}_{X}}=c_{Z,\max}\;\frac{{\mathrm{df}}_{\mathrm{act}}^{\to Z}\left(c_{X}\right)}{{\mathrm{dc}}_{X}}\;\left[1-\text{OR}\left(f_{\mathrm{act}}^{\to Z}\left(\sim X,0\right)\right)\right], \end{array}$$

where ~X means not X and the derivative of the activation function with respect to an input $c_{X}$ is

(A18)
$$\frac{{\mathrm{df}}_{\mathrm{act}}^{\to Z}\left(c_{X}\right)}{{\mathrm{dc}}_{X}}=\mathrm{wB}\left(\frac{{\mathrm{nc}}_{X}^{n-1}}{K^{n}+c_{X}^{n}}-\frac{{\mathrm{nc}}_{X}^{2n-1}}{{\left(K^{n}+c_{X}^{n}\right)}^{2}}\right),$$

and the inhibition function results in

(A19)
$$\frac{{\mathrm{df}}_{\mathrm{inh}}^{\to Z}\left(c_{X}\right)}{{\mathrm{dc}}_{X}}=-\frac{{\mathrm{df}}_{\mathrm{act}}^{\to Z}\left(c_{X}\right)}{{\mathrm{dc}}_{X}}.$$

To examine the derivatives of the demonstrator network output concentration $c_{E}$ w.r.t the inputs A and B, chain rule and backward propagation through the network is used and leads to

(A20)
$$\frac{{\mathrm{dc}}_{E}}{{\mathrm{dc}}_{A}}=\underset{\text{path}\;1}{\underbrace{\frac{{\mathrm{dc}}_{E}}{{\mathrm{dc}}_{C}}\;\frac{{\mathrm{dc}}_{C}}{{\mathrm{dc}}_{A}}}}+\underset{\text{path}\;2}{\underbrace{\frac{{\mathrm{dc}}_{E}}{{\mathrm{dc}}_{D}}\;\frac{{\mathrm{dc}}_{D}}{{\mathrm{dc}}_{A}}}}=-0.832,$$

(A21)
$$\frac{{\mathrm{dc}}_{E}}{{\mathrm{dc}}_{B}}=\underset{\text{path}\;3}{\underbrace{\frac{{\mathrm{dc}}_{E}}{{\mathrm{dc}}_{D}}\;\frac{{\mathrm{dc}}_{D}}{{\mathrm{dc}}_{B}}}}=0.264.$$

This indicates that an increase of $c_{A}$ decreases the concentration $c_{E}$ as the inhibition before the AND connection dominates. But if $c_{B}$ is increased, $c_{E}$ also increases.

### Signaling Network Evaluation

In Figure A4, the time‐dependent solutions for species *Calcium*, *HDAC*, *p38*, *ERK5*, *MEF2*, and *CellArea* are depicted.

In Figure A5, a heatmap is shown illustrating the difference in stationary solutions between the R&M and REF networks. Input concentrations are set to $c_{i}=0.06,\;\forall i\in ℐ$, except for $c_{\text{Stretch}}$, which varies from 0 to 1. The y‐axis displays all species within both networks. Positive values indicate elevated concentrations in the R&M network, while negative values denote higher concentrations in the REF network.

### Asymptotic Stability

The solution of an autonomous system $\dot{c}=f\left(c\right)$ is asymptotically stable if the real parts of all eigenvalues of the Jacobian ${\left(\mathrm{Df}\right)}_{\mathrm{ij}}=\frac{\partial f_{i}}{\partial c_{j}}$ are negative [50]. For the demonstrator network, Df is given as

(A22)
$$Df=\left[\begin{array}{ccc} -\frac{1}{\tau_{C}} & 0 & 0\\ 0 & -\frac{1}{\tau_{D}} & 0\\ \frac{1}{\tau_{E}}\frac{d\text{AND}\left(f_{\mathrm{inh}}^{\to E}\left(c_{C}\right),\;f_{\mathrm{act}}^{\to E}\left(c_{D}\right)\right)}{{\mathrm{dc}}_{C}} & \frac{1}{\tau_{E}}\frac{d\text{AND}\left(f_{\mathrm{inh}}^{\to E}\left(c_{C}\right),\;f_{\mathrm{act}}^{\to E}\left(c_{D}\right)\right)}{{\mathrm{dc}}_{D}} & -\frac{1}{\tau_{E}} \end{array}\right].$$

Thus, the demonstrator network has only negative eigenvalues and therefore the concentrations $c_{i}$ always converge to their stationary solutions independent of the chosen initial conditions. In general acyclic networks, Df has always a lower triangular structure with diagonal entries as its eigenvalues [51]. In case of REF or R&M network, the same problem structure can be observed, that is, the eigenvalues are always $-\frac{1}{\tau_{i}}<0\;\forall i\in S\backslash ℐ$. Therefore, the considered networks are asymptotically stable and their solutions converge to the corresponding stationary values.

### Global Sensitivity Analysis Details

In Figure A6, the first‐ and total‐order sensitivity estimators for the REF and R&M network are depicted. It can be seen that for the chosen sample size of $N_{S}=1\cdot 10^{6}$, all estimators converge to the same solution.

**TABLE A2:** Global sensitivity estimators for *CellArea* with respect to the input species in the REF network.

Species	$S_{\mathrm{jan}}$	$S_{\mathrm{sob}}$	$S_{\mathrm{sat}}$	$T_{\mathrm{jan}}$	$T_{\mathrm{sob}}$	$T_{\mathrm{hom}}$
TNFa	0.0008	0.0007	0.0020	0.0012	0.0013	0.0013
ISO	0.0237	0.0237	0.0250	0.0376	0.0372	0.0372
NE	0.1216	0.1225	0.1240	0.1755	0.1760	0.1760
PE	0.0351	0.0352	0.0367	0.0538	0.0543	0.0543
ET1	0.0218	0.0220	0.0233	0.0329	0.0330	0.0330
AngII	0.1223	0.1225	0.1241	0.1683	0.1681	0.1681
IGF1	0.0843	0.0841	0.0848	0.1162	0.1159	0.1159
EGF	0.0843	0.0838	0.0846	0.1161	0.1156	0.1156
NRG1	0.0841	0.0849	0.0867	0.1160	0.1159	0.1159
TGFb	0.0184	0.0179	0.0196	0.0253	0.0250	0.0250
IL6	0.0435	0.0435	0.0443	0.0607	0.0605	0.0605
FGF	0.0698	0.0701	0.0712	0.0964	0.0964	0.0964
CT1	0.0700	0.0696	0.0699	0.0985	0.0977	0.0977
LIF	0.0683	0.0686	0.0699	0.0983	0.0986	0.0986
Stretch	0.0145	0.0144	0.0159	0.0209	0.0208	0.0208

**TABLE A3:** Global sensitivity estimators for *CellArea* with respect to the input species in the R&M network.

Species	$S_{\mathrm{jan}}$	$S_{\mathrm{sob}}$	$S_{\mathrm{sat}}$	$T_{\mathrm{jan}}$	$T_{\mathrm{sob}}$	$T_{\mathrm{hom}}$
TNFa	0.0007	0.0008	0.0021	0.0012	0.0012	0.0012
ISO	0.0200	0.0202	0.0214	0.0345	0.0342	0.0342
NE	0.1016	0.1026	0.1038	0.1612	0.1618	0.1618
PE	0.0294	0.0297	0.0311	0.0494	0.0500	0.0500
ET1	0.0186	0.0189	0.0202	0.0304	0.0305	0.0305
AngII	0.1024	0.1036	0.1052	0.1555	0.1559	0.1559
IGF1	0.0933	0.0929	0.0935	0.1382	0.1377	0.1377
EGF	0.0731	0.0726	0.0733	0.1099	0.1097	0.1097
NRG1	0.0728	0.0739	0.0763	0.1100	0.1101	0.1101
TGFb	0.0162	0.0159	0.0176	0.0242	0.0240	0.0240
IL6	0.0374	0.0377	0.0385	0.0570	0.0569	0.0569
FGF	0.0610	0.0612	0.0624	0.0916	0.0917	0.0917
CT1	0.0599	0.0597	0.0601	0.0915	0.0907	0.0907
LIF	0.0583	0.0586	0.0597	0.0914	0.0917	0.0917
Stretch	0.0772	0.0771	0.0787	0.1187	0.1189	0.1189
